# Prevalence of Molar-incisor hypomineralization in Iranian children – A systematic review and narrative synthesis

**DOI:** 10.1038/s41405-022-00111-x

**Published:** 2022-06-13

**Authors:** Elham Afshari, Farzaneh Dehghan, Mohammad Ali Vakili, Marzieh Abbasi

**Affiliations:** 1 Department of community Oral Health, School of Dentistry, Golestan University of Medical Science, Golestan, Gorgan, Iran; 2 Department of Pediatric Dentistry, School of Dentistry, Golestan University of Medical Science, Golestan, Gorgan, Iran; 3 Health Management and Social Development Research Center, Department of Health and Community Medicine, Faculty of Medicine, Golestan University of Medical Sciences, Golestan, Gorgan, Iran; 4 Department of Pediatric Dentistry, School of Dentistry, North Khorasan University of Medical Science, Bojnord, North Khorasan Iran

**Keywords:** Paediatric dentistry, Restorative dentistry

## Abstract

**Introduction:**

Molar-incisor hypomineralization is a developmental defect of enamel with clinical features vary from demarcated opacities to severe tissue breakdown which calls for considerable preventive and interceptive measures. The aim of this article was to systematically review the literature on the prevalence of MIH in Iran and highlight the condition in Iranian children.

**Materials and Methods:**

A systematic search of literature was conducted in Scopus, Pubmed, Ovid, Embase, Web of Science, and Google-Scholar as well as national Iranian database and digital archives of dental schools from the beginning of 2000 to the end of 2021 for published and unpublished studies. Data from cross-sectional, cohort, and case-control studies on prevalence of molar-incisor hypomineralization among 6–13-year-old children was gathered, using the following MeSH terms and keywords and their Persian equivalents: Prevalence, Hypomineralisation, Hypomineralization, MIH, “molar incisor”, “molar-incisor”, “cheese molars”, “Hypomineralised first permanent molars”, “Hypomineralized first permanent molars”, “developmental defects of enamel”, “enamel developmental defects”, Iran*. Methodological quality and the risk of bias of quantitative studies was assessed using a modified version of Newcastle-Ottawa Scale. Due to the considerable clinical and statistical heterogeneity of the included studies, pooling of data through meta-analysis was not possible. Therefore, a descriptive synthesis of data was performed.

**Results:**

Fifteen cross-sectional studies with a total number of 12011 participants were included in the systematic review. The prevalence of MIH ranged from 5.1% to 25.6%. All of the included studies were at a moderate risk of bias (NOS of 4-6). The lowest prevalence of MIH was reported in Kerman (5.14%) and the highest in Tehran (25.6%). Substantial methodological, clinical and statistical heterogeneity was observed.

**Conclusion:**

This is the first study to systematically review the available literature on MIH prevalence in Iran. However, the present review has some limitations such as limited number of included studies, large heterogeneity of the research, and moderate quality of included studies. Further high-quality research is warranted.

## Introduction

The term “Molar-Incisor Hypomineralization (MIH)” was first introduced by Weerheijm in 2001 [1] which refers to hypomineralization of systemic origin which occurs in one to four first permanent molars and frequently affects incisors as well [2]. Clinical presentations vary from demarcated opacities and enamel disintegration to atypical restorations and extracted teeth [3]. The most frequently-reported clinical issues associated with MIH include enamel surface breakdown, hypersensitivity, difficulties in achieving local anesthesia, behavioral management problems, anesthetic problems, tooth loss, eruption difficulties, negative impact on child’s school performance, and financial concerns [4].

Although the exact etiology of MIH remains uncertain, different factors have been thought to be implicated in development of the defects, such as prenatal and perinatal adverse events, early childhood illnesses, early childhood medications, breastmilk digoxin, and genetic predisposition and epigenetic influences [5].

During the last decades, different indices and diagnostic criteria have been used to conduct studies on MIH, including the European Archives of Pediatric Dentistry (EAPD) diagnostic criteria [6], the developmental enamel defects (DDE) index presented by FDI [7], and the modified DDE (mDDE) index [8]. Several attempts have also been made to classify the severity of MIH according to clinical features or sensitivity of the affected teeth [9].

The prevalence of MIH varies in different countries and regions. A previous systematic review in 2018, estimated a 14.2% global prevalence of MIH, with the highest and lowest prevalence in South America (18.0%) and Africa (10.9%) respectively [10]. A more recent meta-analysis published in 2021, reported a pooled prevalence of 13.5%, with the highest and lowest prevalence in American (15.3%) and Asian (10.7%) continents [11]. Other studies have even reported prevalence as low as 2.7% (Egypt) [12] to up to 19.7% (Brazil) [13].

Knowing the prevalence of the condition, assists policymakers in planning the appropriate public oral healthcare strategies. Having this in mind and the realization of regional variations in prevalence, performing country-specific studies is of high importance. Many researchers have investigated the prevalence of MIH across different regions of Iran. However, the studies are spare and need to be systematically reviewed. Therefore, the current study aimed to systematically review the studies describing prevalence rates of MIH in Iranian children, to assess the methodological issues of the included studies, and to prepare recommendations for future research.

## Materials and methods

### Search strategy

This systematic review was designed and reported within the PRISMA framework [14] – the study protocol was not published, but is available on request. The study protocol was registered and approved by Golestan University of Medical Sciences (No: 27-111122). A comprehensive search was performed in international databases of Scopus, Pubmed, Ovid, Embase, Web of Science, as well as national Iranian database of Magiran, SID, and IranDoc in order to review the studies on MIH prevalence in Iranian children from the 1 January of 2000 to the 31 December of 2021. Furthermore, the national database of medical sciences dissertations and theses (http://thesis.research.ac.ir/) and digital archives of dental schools were searched for unpublished data and supplementary search of Google-Scholar and manual search of reference lists of included studies were also conducted.

Search terms included the following MeSH terms and keywords and their Persian equivalents: Prevalence, Hypomineralisation, Hypomineralization, MIH, “molar incisor”, “molar-incisor”, “cheese molars”, “Hypomineralised first permanent molars”, “Hypomineralized first permanent molars”, “developmental defects of enamel”, “enamel developmental defects”, “Iran”.

### Study selection

All studies were independently reviewed by two investigators (FD and EA) for eligibility via title and abstract, and then as full-text. As planned in the study protocol, in case of discrepancies between the two reviewers, a third author (AV) had to be consulted. However, no discrepancies were noted.

The PICO (patients, intervention, comparator and outcome) for studies’ selection was as follows:

- Patients: six-to 12-year-old Iranian children
- Intervention: Intervention was replaced with the “phenomenon of interest” which was MIH
- Comparator: Not applicable
- Outcome: Prevalence of MIH

The inclusion criteria are listed as follows:

1. Study type: cross-sectional, cohort, and case-control
2. Study language: English and/or Persian
3. Study population: including 6–12 year-old Iranian children
4. Population-based studies where the prevalence of MIH was assessed through clinical examinations

The exclusion criteria were as follows:

1. Studies on specific populations (such as children with celiac disease or asthmatic conditions)
2. Studies with incomplete data or if the data could not be obtained from the authors
3. Studies with duplicate data
4. Studies with sample size less than 100

### Data extraction

The following data was sought from the eligible studies independently by two investigators (FD and EA): title, first author’s name, region of sample origin, year of publication (if published), year in which the study was conducted, type of study, diagnostic criteria/indices, age of participants, number and sex of participants, prevalence of MIH. In case of insufficient data or unpublished studies, the authors were contacted for additional data.

### Risk of bias and quality assessment

The included studies were evaluated by one reviewer, using a modified version of the Newcastle-Ottawa scales (NOS) adapted for cross-sectional studies [15]. The scale covers the following domains: Selection of participants, sample size justification, outcome measurement, and confounding adjustment. A score (0%3) was attributed to each grade (A to D) (appendix 1). The scores were summed up to form a total score between 0 and 8. Study was considered as displaying a high risk of bias (i.e., low quality) when NOS was ≤3, moderate risk of bias (i.e., moderate quality) when NOS was 4–6, and low risk of bias (i.e., high quality) when NOS was ≥7.

### Data synthesis

Due to the considerable clinical heterogeneity — namely differences in participants (e.g., age, gender), diagnostic criteria (DDE, EAPD), and the assessment method— and substantial statistical heterogeneity of the included studies (I^2^ value of 95.25%), a meta-analysis was not performed. Thus, a descriptive synthesis of evidence was performed. Studies which their primary goal was not to assess the prevalence of MIH in random population were also included if prevalence rates could be extracted.

## Results

### Literature search and

**Fig. 1:**
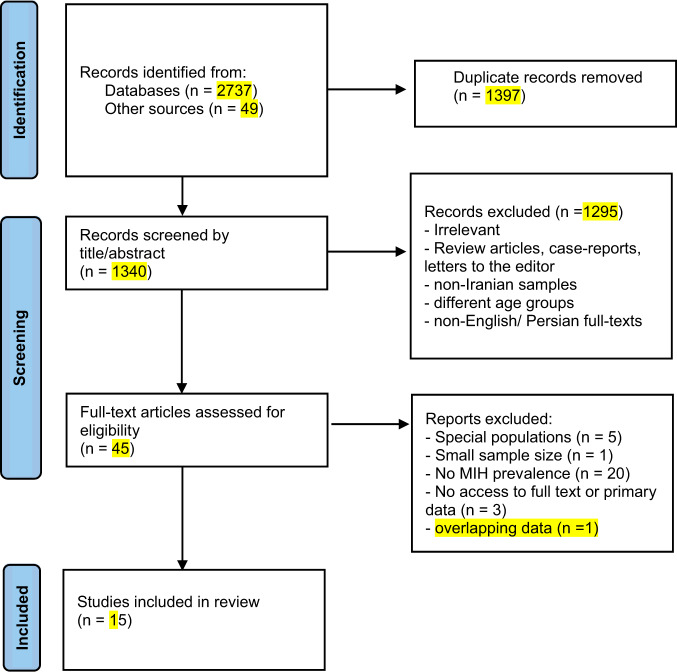
PRISMA Flow chart. The PRISMA flow diagram for the systematic review detailing the study screening and selection process including number of studies screened, number of excluded and retrieved studies, and the reasons for exclusion.

The flow chart for study selection is presented in Fig. 1. The initial search resulted in 2737 titles from databases and 49 from other sources. Of 45 articles reviewed in full-text, most were excluded because they did not report the prevalence rates. Five articles were excluded because they focused on special populations. One study was excluded because of overlapping data [16].

Providing the prevalence rate of MIH could be extracted, studies with a primary outcome rather than prevalence of MIH in random population were also included. Only one study was excluded because of small sample size [17]. Fifteen studies met the final inclusion criteria for the review [18–32].

### Characteristics of included studies

Description of the included studies is shown in Table 1. The total sample size in 15 included studies (9 published and 6 unpublished) was 12011 participants. The included studies originated from 13 geographic areas. All of the included studies were performed in school-based settings. Published studies were published between 2012 and 2021; and unpublished studies which all were dissertations or theses, were conducted between 2015 and 2019.

**Table 1:** Characteristics of the included studies.

First author	region	Sample	Year of publication	Year of conduct	Study type	Diagnostic criteria	Prevalence of MIH %
Ahmadi et al. [18]	Zahedan	433 children aged 7–9	2012	2011	Cross-sectional	DDE	12.7
Ghanim et al. [19]	Shiraz	810 children aged 9–11	2014	2013	Cross-sectional	EAPD	20.2
Salem et al. [20]	Masal-Shanderman	553 children aged 6–13	2016	2015–2016	Cross-sectional	EAPD	18.4*
Salem et al. [21]	Rasht	1043 children aged 6–13	2017	2016	Cross-sectional	EAPD	19.93
Bahrololoomi et al. [22]	Yazd	645 children aged 7–11	2017	2016	Cross-sectional	EAPD, mDDE	23.87
Poureslami et al. [23]	Kerman	779 children aged 7–12	2018	2015–2016	Cross-sectional	EAPD	6.5
Karimi et al. [24]	Kermanshah	1081 children aged 8–12	unpublished	2015	Cross-sectional	EAPD	9.5
Salari et al. [25]	Tehran	1028 children aged 7–12	unpublished	2016–2017	Cross-sectional	EAPD	25.6
Karimi et al. [26]	Kerman	501 children aged 8–12	unpublished	2018	Cross-sectional	EAPD	8.4
Moshfeghnia et al. [27]	Yasooj	568 children aged 7–12	unpublished	2018	Cross-sectional	EAPD	10.8
Rezayee et al. [28]	Bojnourd	474 children aged 7–9	unpublished	2018–2019	Cross-sectional	EAPD	14.3
Kaffashchian et al. [29]	Tabriz	369 children aged 8–10	unpublished	2019	Cross-sectional	EAPD	10.84
Einollahi et al. [30]	Ardabil	520 children aged 8–10	2020	2019	Cross-sectional	EAPD	24
Shojaeepour et al. [31]	Kerman	2507	2020	2019	Cross-sectional	EAPD	5.14
Hali et al. [32]	Sari	700 children aged 7–12	2021	2019	Cross-sectional	DDE	20.2

^*^The total prevalence reported in the article did not match the other data, so it was recalculated using raw data.

Of the 15 included studies, 9 were written in Persian and 6 were written in English. All included studies were cross-sectional. Among the eligible studies, two used DDE diagnostic criteria, one used a combination of EAPD and mDDE and the rest used EAPD diagnostic criteria. The sample size of included studies ranged from 369 to 2507.

### Quality assessment of included studies

The included studies were evaluated using a modified version of the NOS adapted for cross-sectional studies. The scale produces a score between 0 and 8. Study was considered as high quality when NOS was 7–8, moderate quality when NOS was 4–6, and low quality when NOS was 3 and less. All of the included studies were scored as moderate quality (NOS of 4–6).

### Prevalence by geographical region

**Fig. 2:**
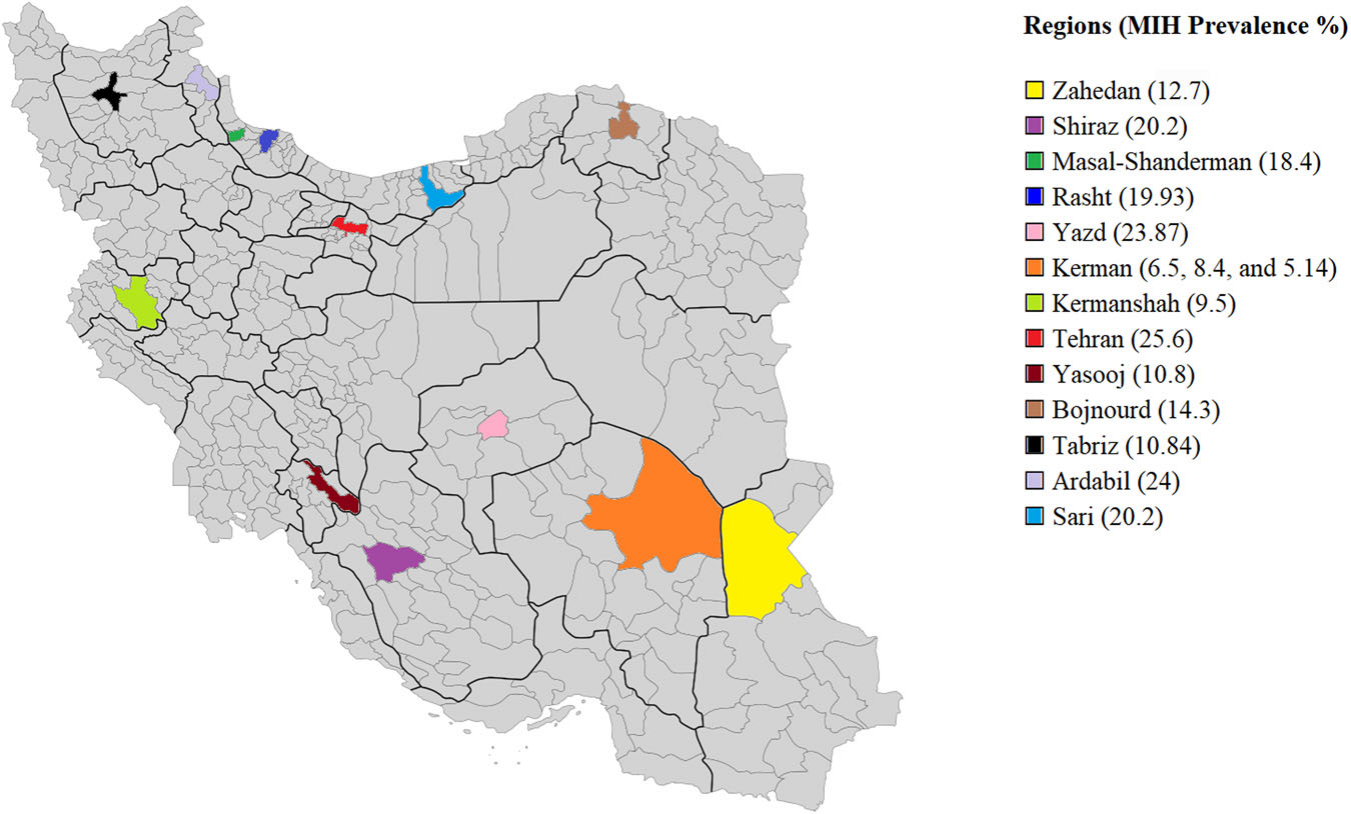
Prevalence of MIH in Iran. Map highlighting the regional distribution of prevalence of MIH in Iran based on the records retrieved in the systematic review.

There was substantial variation in reported MIH prevalence rates. Of the 13 studied geographical regions, the lowest prevalence of MIH was detected in Kerman [31] (5.14%) and the highest in Tehran [25] (%25.6). Kerman was the most studied region with prevalence rates of 6.5%, 8.4%, and 5.14% in three studies [23, 26, 31] Fig. 2.

### Prevalence by sex

Twelve studies compared prevalence rates in two genders [18–21, 23–25, 27–30, 32]. Two study reported that MIH lesions were seen more often in girls [19, 24] while others reported no significant difference of prevalence between two genders. One study focused only on girls [26], while others included both genders.

### Prevalence by tooth type and dental arch

Of the 15 included studies, 10 assessed the prevalence of MIH according tooth type [18, 19, 21, 23–25, 27, 28, 31, 32]. One study reported the upper right molars and central incisors as the most frequently affected teeth [19], while others reported that molars were more affected than incisors. Two studies reported no significant difference in the MIH prevalence in lower and upper arches [19, 28]. One study reported that prevalence of MIH was higher in the lower arch [23], while 2 other studies study reported the lesions to be more prevalent in the upper arch [24, 27]. one of the included studies reported that lower molars were more affected than upper molars, while upper incisors were more affected than lower ones [21]. Finally, one study declared that no significant difference was revealed in the prevalence of affected molars by dental arch, while incisors were more affected in the upper arch [21].

### Prevalence by age

Eight of the included studies reported MIH prevalence according to age [19–21, 24, 25, 28, 29, 31], most of which showed no specific trend [19, 20, 25, 28, 29]. Two studies showed a higher prevalence with increasing age [21, 24]. Also MIH prevalence according to age groups was reported in one study but not analyzed statistically, though lack of specific trend with increasing age was observed from presented data [31].

### Other notable findings

#### Dental caries

As logically anticipated, DMFT was reported to be significantly higher in children affected with MIH than the control groups in most studies [18, 22, 26, 28]. Also one study, though not using DMFT index, declared that more children had dental caries in MIH group than the control group 29. However, surprisingly one study showed a reverse relationship between prevalence of MIH and DMFT [24].

#### Etiologic factors

Of the studies reviewed, nine assessed the etiological factors of MIH. According to the obtained data, history of the following factors had statistically significant impact on MIH prevalence:

- Prenatal and perinatal: Pregnancy complications [18], persistent fever during pregnancy [26], type of delivery [18, 25, 27, 28], birth complications [18, 25], hypoxia during delivery [26], premature birth [18, 29], medicine use during pregnancy [29], smoking during pregnancy [29], Use of amoxicillin during pregnancy [29], and maternal diabetes mellitus [27].
- Postnatal: Ear infection [18], chicken pox [18, 25, 29], renal failure [18], allergies [18], amoxicillin usage [18, 25], Birth delivery type [25, 27, 28], breastfeeding over 12 months [18, 20, 27, 29], urinary tract infection [25, 29], high fever [26, 30], jaundice and exchange transfusion 29, breast-milk allergy [29], asthma and other respiratory diseases [29, 30], allergy [30], cleft lift and palate [29], vitamin deficiency [29], history of diarrhea and vomiting [29], history of hospitalization [30]. One of the reviewed studies did not find a significant association between any of the prenatal, perinatal, and postnatal factors on the MIH prevalence [21]

#### body mass index (BMI) and body weight

Four studies assessed the association of MIH and BMI or body weight [19, 22, 26, 28]. One of these studies showed a negative relationship between obesity and MIH [19], one study showed no relationship between BMI and MIH prevalence [22]. Another study declared that MIH prevalence was higher in low weight children [26]. And finally, one study showed no statistically significant relationship between MIH prevalence and BMI in general population of children or in girls, while the condition was more prevalent in boys with normal BMI [28].

## Discussion

MIH is a prevalent dental defect around the world, placing the highest burden (e.g., economic, esthetic, psychologic) on low- and middle-income countries [29]. To the best of our knowledge, the current study is the first systematic analysis of the MIH prevalence in Iran. Our data analyzed 12011 participants in 15 studies from 2012 to 2019. Generalizability of the findings might be limited do to the high heterogeneity of the included studies.

In this systematic review, the lowest and highest prevalence rates were reported by studies conducted in Kerman (5.14%) [31] and Tehran (25.6%) [25] respectively. The second lowest prevalence was again reported by another study in Kerman (6.5%) [23]. The large variations may be explained by several factors such as ethnic and environmental variations and methodological differences. Although almost all studies used the same EAPD diagnostic criteria, two studies used DEE [18, 22] and one used a combination of EAPD and mDDE criteria [32]. The operator dependency of visual diagnosis may also contribute to variations. Probably due to the same mentioned reasons, reported prevalence of MIH across the globe shows a wide variation (2.4–40.2%) as well [22].

Of the 15 included studies, all showed moderate quality/risk of bias. “Selection bias” was the main source of bias, followed by “assessment of outcome”. All of the included studies were performed in the school settings. The random sampling procedures when selecting the schools were not explained or were impaired in some studies, therefore, the samples may not be representative of the population.

Most of the included studies reported no significant difference of prevalence between two genders [18, 20, 21, 23, 25, 27–30, 32] which in in accordance with the results reported by other systematic reviews [11, 33]. However, two reported that MIH was more prevalent in girls [19, 24] which can be attributed to the difference in time of eruption between two genders [34].

Almost all of the included studies reported that molars were more affected than incisors which agrees with the results from other populations [35–37]. However, variation exists within the included studies according to the most affected dental arch. The mentioned variation also in studies conducted in other countries, with the upper arch being more affected in some studies [38–40], the lower arch in some studies [36, 41], and no difference in some others [37, 42, 43].

Although the prevalence rates differed between age groups in the included studies, most showed no trend relating prevalence to age [19, 20, 25, 28, 29]. A global systematic review [10] which showed that the prevalence of MIH among children 10 years of age or younger was much higher than older children. However, data from the mentioned review also showed no trend.

Considering the variation and heterogeneity of studies, more standardized high-quality studies are needed to provide the data necessary for a meta-analysis. Although the mentioned variations make it difficult to make a precise comparison, the overall prevalence and regional rates of MIH in Iran assessed in the current study seem to be high which brings up the need for improving the practical skills of oral health care workers in early diagnosis and management of the condition. Studies have shown that children suffering MIH are about 4–10 times more likely to undergo treatment for their permanent first molars [44, 45]. Early diagnosis makes it possible to manage the condition using preventive and conservative interventions such as repeated application of fluoride varnishes, GIC restorations, and fissure sealants [46, 47], which are less invasive, cheaper, easier, and cause less pain and discomfort to the patients compared to the treatments such as full metal crown or tooth extraction which are needed after severe tissue breakdown that happens rapidly in cases of late diagnosis and treatment [46, 48].

## Limitations

Although this is the first study to systematically review the studies on the prevalence of MIH in Iran, the limitations of the present study should be acknowledged. First, the results should be interpreted by caution due to the limited number of included studies and large heterogeneity. Second, although every effort was made to contact the authors regarding full text of studies and raw data, several relevant studies were excluded because of lack of such access. Third, all of the included studies were of moderate quality and were not selected on basis of quality criteria because of the lack of high-quality studies.

## Conclusion

This is the first study to systematically review the available literature on MIH prevalence in Iran. However, the present review has some limitations such as limited number of included studies, large heterogeneity of the research, and moderate quality of included studies. Further high-quality research is warranted.

## Supplementary information

Appendix 1
