# Supplementary material for: Prevalence of Molar-incisor hypomineralization in Iranian children – A systematic review and narrative synthesis
Source: BDJ Open. 2022 Jun 13;8:15. doi: 10.1038/s41405-022-00111-x (PMC9192646; doi:10.1038/s41405-022-00111-x)
Supplement: Supplementary file 1 — Appendix 1 [file 41405_2022_111_MOESM1_ESM.docx]

|  | | Ahmadi et al. ^18^ | Ghanim et al. ^19^ | Salem et al. ^20^ | Salem | Bahrololoomi | Poureslami et al. ^23^ | Karimi et al. ^24^ | Salari et al.^25^ | Karimi et al.^26^ | Moshfeghnia et al. ^27^ | Rezayee et al.^28^ | Kaffashchian et al.^29^ | Einollahi et al.^30^ | Shojaeepour et al.^31^ | Hali et al.^32^ |
| --- | --- | --- | --- | --- | --- | --- | --- | --- | --- | --- | --- | --- | --- | --- | --- | --- |
| Selection (representativeness of the sample) | A. All subjects or random sampling (3)  B. Non-random sampling (2)  C. Selected group of users (1)  D. No description of sampling strategy (0) | 1 | 3 | 1 | 2 | 2 | 1 | 2 | 2 | 3 | 2 | 2 | 3 | 3 | 2 | 3 |
| Selection (sample size) | A. Justified and satisfactory (1)  B. Not justified (0) | 0 | 0 | 0 | 1 | 1 | 1 | 1 | 1 | 0 | 1 | 0 | 0 | 1 | 1 | 0 |
| Detection (outcome measurement) | A. Validated measurement tool (2)  B. Tool described but non-validated (1)  C. Tool not described (0) | 2 | 2 | 2 | 0 | 2 | 2 | 1 | 0 | 1 | 2 | 2 | 2 | 1 | 2 | 2 |
| Confounding | A. Adjusted for confounders (1)  B. No adjustment for confounders (0) | 1 | 1 | 1 | 1 | 0 | 1 | 0 | 1 | 0 | 0 | 1 | 0 | 1 | 1 | 0 |
| Detection (Outcome assessment) | A. Independent blind assessment (1)  B. Record linkage (0)  C. Self-report (0)  D. No description (0) | 0 | 0 | 0 | 0 | 0 | 0 | 0 | 0 | 0 | 0 | 0 | 0 | 0 | 0 | 0 |
| Total score |  | 4 | 6 | 4 | 4 | 5 | 5 | 4 | 4 | 4 | 5 | 5 | 5 | 6 | 6 | 5 |

Appendix 1: Modified Newcastle-Ottawa quality assessment scale for included studies
